# The distinct role of NR2B subunit in the enhancement of visual plasticity in adulthood

**DOI:** 10.1186/s13041-015-0141-y

**Published:** 2015-08-19

**Authors:** Hanxiao Liu, Yue Li, Yan Wang, Xinxing Wang, Xu An, Siying Wang, Lin Chen, Guosong Liu, Yupeng Yang

**Affiliations:** Chinese Academy of Sciences Key Laboratory of Brain Function and Diseases, and School of Life Sciences, University of Science and Technology of China, Hefei, 230027 China; Hefei National Laboratory for Physical Sciences at the Microscale, University of Science and Technology of China, Hefei, 230027 China; School of Basic Medicine, Anhui Medical University, Hefei, 230032 China; Tsinghua-Peking Centre for Life Sciences, School of Medicine, Tsinghua University, Beijing, 100084 China

## Abstract

**Background:**

Experience-dependent plasticity is confined to the critical period of early postnatal life, and declines dramatically thereafter. This attenuation promotes the stabilization of cortical circuits, but also limits functional recovery of several brain diseases. The cognitive functions and synaptic plasticity in the hippocampus and prefrontal cortex are elevated following chronic magnesium treatment. Here, we explored the effect of magnesium treatment on visual plasticity and the potential clinical significance.

**Results:**

Visual plasticity in adult mice was dramatically enhanced following magnesium treatment, which was concurrent with an increase in the expression of NR2 subunits of *N*-methyl-D-aspartate receptors. Blockade of NR2B activity in both the induction and expression periods of plasticity prevented this reinstatement. However, the plasticity restored *via* a decrease in cortical inhibition was independent on the activation of NR2B, indicating a different underlying mechanism. The functional excitatory synapses on layer 2/3 pyramidal neurons were increased following magnesium supplementation. Moreover, the synaptic and neuronal responses were reminiscent of that within the critical period, and this rejuvenation of adult visual cortex facilitated the recovery of visual functions in amblyopia.

**Conclusions:**

Collectively, our data reveal two distinct mechanisms underlying the restoration of visual plasticity in adulthood, and the rejuvenation of adult visual cortex following magnesium treatment provides a new avenue to develop clinical therapies for adult amblyopia, as well as to explore plasticity-based treatment of other brain diseases, such as stroke and aphasia.

**Electronic supplementary material:**

The online version of this article (doi:10.1186/s13041-015-0141-y) contains supplementary material, which is available to authorized users.

## Background

The rewiring of neural circuits with external experience is a fundamental property of the central nervous system. However, due to the formation of the functional and structural barriers, this capability diminishes in the sensory cortex following the critical period of postnatal development [1–4]. This attenuation restricts potential therapy for numerous brain diseases [5, 6]. Therefore, the reinstatement of plasticity in the adult cortex is not only an important scientific question regarding the maturation of neural circuitry, but also a central issue in the development of effective therapies for brain diseases.

Visual cortex is the most classic region for studying experience-dependent plasticity. NR2A and NR2B are two predominant NR2 subunits of *N*-methyl-D-aspartate receptors (NMDARs) in the visual cortex. A developmental switch from NR2B to NR2A subunits of NMDARs, in addition to a decrease in spine density, occurs concurrently with the decline in visual plasticity [7–9]. Since the critical period is normally terminated in NR2A-deficient mice [10], the increased incorporation of NR2A could not attribute to the closure of the critical period. In contrast to NR2A-containing NMDARs, NR2B-containing NMDARs with slow kinetics of deactivation mediate higher levels of calcium entry through ion channels and exhibit a higher affinity for calcium/calmodulin-dependent protein kinase II, both of which are essential for visual plasticity [11, 12]. Considering its contribution on synaptogenesis and synaptic efficacy [13, 14], NR2B-containing NMDARs may be important to the reinstatement of plasticity in adulthood [15, 16].

Due to the lethality of NR2B knockout mice and the failure of NR2B overexpressing in the visual cortex of transgenic mice, the contribution of NR2B to visual plasticity is still obscure [17, 18]. Recent studies show that the upregulation of NR2B and synaptogenesis can be synchronously induced in the hippocampus and prefrontal cortex of rodents by a chronic elevation in the concentration of magnesium in the brain [19, 20]. In the present study, we demonstrated that magnesium treatment reinstated visual plasticity in adult mice, which relied on the activation of NR2B-containing NMDARs. Interestingly, NR2B activation was not prerequisite for the restoration of visual plasticity by a reduction in cortical inhibition. Both neural responses and synaptic transmission in the visual cortex presented a juvenile-like property following magnesium treatment, which facilitated the full recovery of behavioral acuity and neural functions from adult amblyopia. Therefore, we believed that magnesium supplementation deserves further investigation as an adjuvant, non-invasive clinical therapy for adult amblyopia.

## Results

### The restoration of juvenile-like OD plasticity in adult mice

**Fig. 1:**
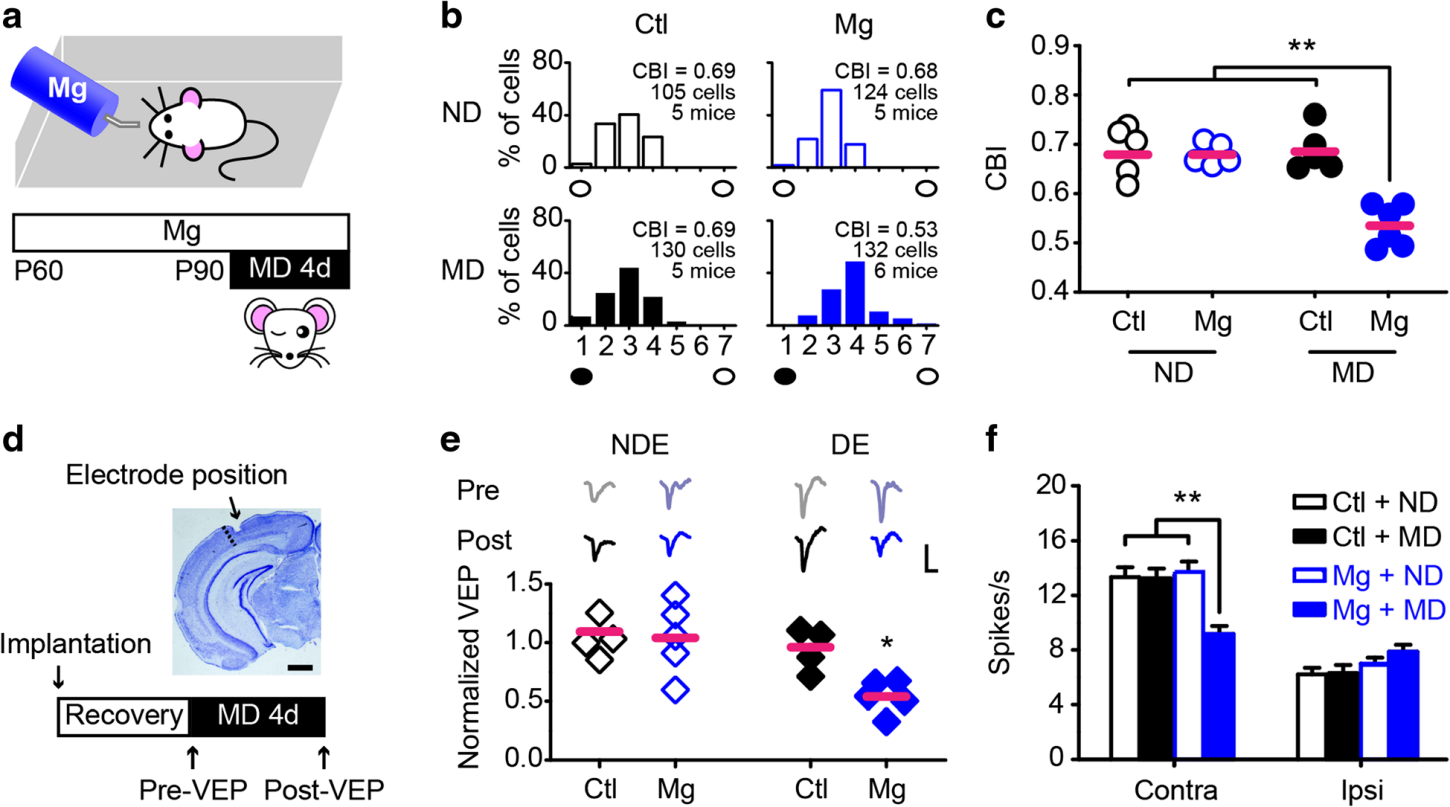
Restoration of juvenile forms of visual plasticity in adult mice following magnesium treatment. **a** Schematic of the experimental procedure. **b** OD distribution for adult control (Ctl, left column) and magnesium-treated (Mg, right column) mice with (MD) or without (ND) monocular deprivation. The number of cells and animals per group is as indicated. Filled black circles represent the deprived eye, and open black circles represent the non-deprived eye. **c** A summary of the CBIs of each group shown in (**b**). **d** The position of the implanted microelectrode and a schematic of the experimental procedure. The dashed line represents the border between V1 and V2. Scale bar, 1 mm. **e** (top) Representative traces showing the VEPs of each eye recorded before (Pre) or after (Post) 4 days of MD. Scale bars, 100 μV and 100 ms. (bottom) Summary of the effects on the responsiveness in each eye induced by MD. The post-VEP amplitude was normalized to the pre-VEP amplitude of the identical eye. DE, deprived eye; NDE, non-deprived eye. *P = 0.03 using paired Student’s *t* test. **f** Stimulation-evoked firing rates for each eye in groups shown in (**b**). Contra, contralateral eye; Ipsi, ipsilateral eye. Error bars, SEM. In **c**, **e**, the horizontal bar represents the mean value; each symbol represents one animal. In **e**, **f**, **P < 0.01 using a one-way ANOVA followed by Tukey’s *post hoc* test

Adult C57BL/6 mice were randomly divided into two groups: an experimental group that was supplied with magnesium *via* drinking water, and a control group that was provided with normal water for one month. Ocular dominance (OD) plasticity was measured in the entire thickness of binocular zone of the primary visual cortex (V1b) following 4 days of monocular deprivation (MD) (Fig. 1a). In adult mice receiving normal drinking water, the OD distribution favored the contralateral eye, and this preference was impervious to MD (Fig. 1b), which is consistent with previous studies [21, 22]. In contrast, mice in the magnesium-treated group exhibited a robust OD shift toward the open ipsilateral eye following MD (Fig. 1b). Magnesium itself did not affect the OD distribution, the mean firing rates of the visually evoked and spontaneous activities, or the body weight (Fig. 1b, Additional file 1: Figure S1). Both the contralateral bias index (CBI, Fig. 1c) and the cumulative distribution of the OD score (Additional file 1: Figure S2a) were significantly different in the deprived magnesium-treated group compared with the remaining groups, suggesting a restoration of visual plasticity.

A loss of responsiveness in the deprived eye after short-term MD typically indicates a juvenile visual plasticity [23, 24]. We implanted a microelectrode into layer IV (400 μm below the brain surface) of V1b and recorded the visually evoked potential (VEP) in individual mice prior to (pre-VEP) and following (post-VEP) 4 days of MD (Fig. 1d). As expected, the contralateral-to-ipsilateral (C/I) VEP ratio was significantly reduced in magnesium-treated mice, while barely altered in normal adult mice following MD (Additional file 1: Figure S2b). We further normalized the post-VEP amplitude to the pre-VEP amplitude of the identical eye. In control mice, the VEP amplitudes of both eyes were constant, indicating a lack of visual plasticity (Fig. 1e). In contrast, we found a significant reduction in the VEP amplitude of the deprived eye without changes in the non-deprived eye in magnesium-treated mice (Fig. 1e). Consistent with these findings, single-unit recordings indicated that MD induced a decrease in the mean firing rate of the deprived eye in magnesium-treated mice (Fig. 1f). These results demonstrate a juvenile-like property of the restored plasticity.

### NR2B-dependent restoration of visual plasticity following magnesium treatment

**Fig. 2:**
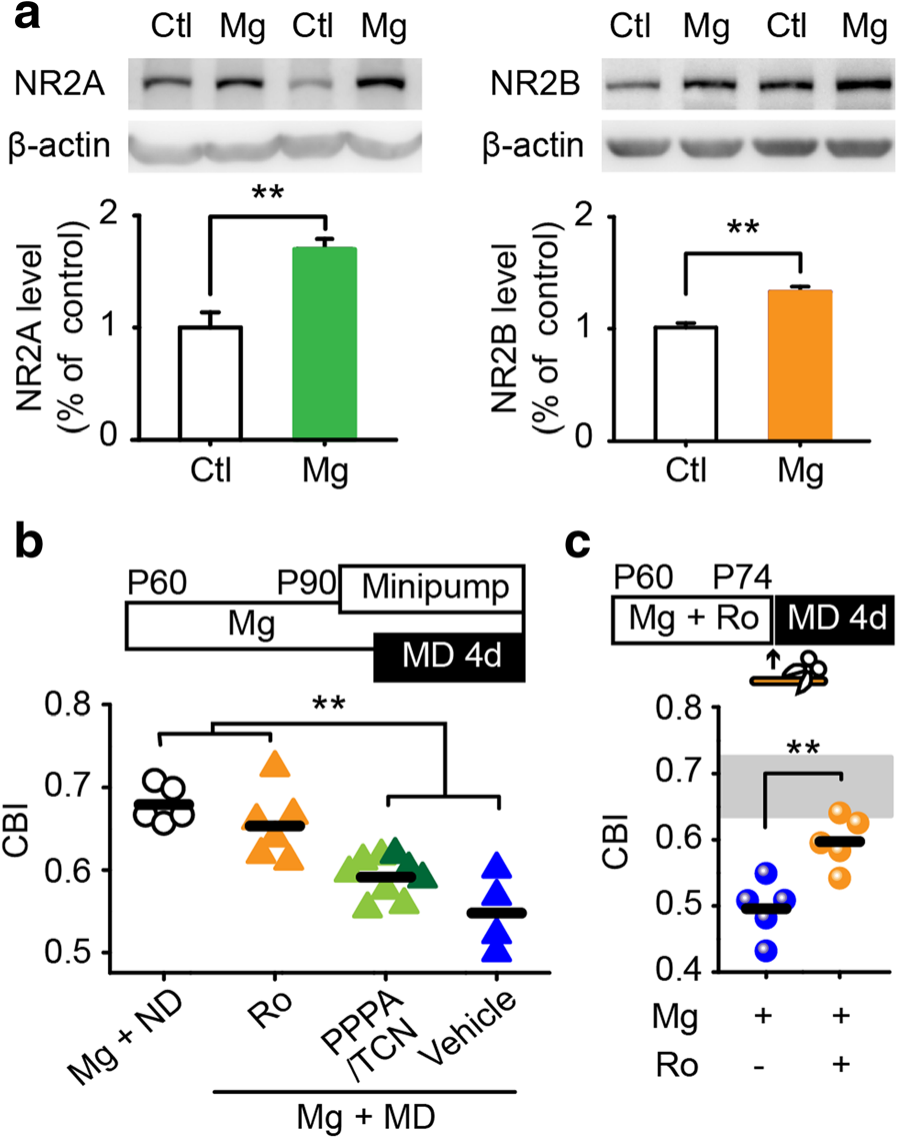
The crucial role of NR2B-containing NMDARs in the magnesium-induced restoration. **a** The protein expression levels of NR2A (Ctl, 11 mice; Mg, 13 mice) and NR2B (Ctl, 12 mice; Mg, 13 mice) in V1b of control and magnesium-treated mice. Using two-tailed Student’s *t* test, P = 0.0002 for NR2A, P = 0.00002 for NR2B. Error bars, SEM. **b** Schematic of the experimental procedure (top) and a summary of the CBIs in each group with a local infusion of NR2B antagonist (Ro, Ro 25-6981), NR2A antagonists (PPPA/TCN; PPPA, light green; TCN 201, dark green) or vehicle (bottom). The drugs were locally infused to V1b of magnesium-treated mice using an osmotic minipump one day prior to MD. P < 0.01 using a one-way ANOVA followed by Tukey’s *post hoc* test. **c** Schematic of the experimental procedure (top) and a summary of the CBIs in magnesium-treated mice with or without Ro 25-6981 (bottom). Both magnesium and Ro were removed prior to the onset of MD. The gray area represents the normal CBI level. The horizontal bar represents the mean value; each symbol represents one animal. P = 0.005 using two-tailed Student’s *t* test. **P < 0.01

NMDAR-mediated signaling is one of the most important signaling pathways involved in cortical plasticity [12]. We found that the protein levels of both the NR2A and the NR2B subunits in V1b were significantly higher in magnesium-treated mice compared with those in control mice (Fig. 2a), while the NR2A/NR2B ratio was not affected by magnesium treatment (Additional file 1: Figure S3a). A systemic (intraperitoneal) injection of the NMDAR antagonist MK801 prevented OD plasticity in magnesium-treated mice (Additional file 1: Figure S3b, c). To further examine the contributions of the NR2A and NR2B subunits, we locally infused the NR2A antagonists PPPA or TCN 201, the NR2B antagonist Ro 25-6981, or vehicle into the visual cortex of magnesium-treated mice using an osmotic minipump one day prior to and throughout 4 days of MD (Fig. 2b). Strikingly, the OD shift was entirely blocked by NR2B antagonist, but not by NR2A antagonists or vehicle (Fig. 2b, Additional file 1: Figure S4a).

To further exclude the possibility that NR2B subunits merely participate in the expression but not the restoration of plasticity, we simultaneously administered magnesium and the NR2B antagonist Ro 25-6981 for 2 weeks, followed by the removal of both prior to the onset of MD (Fig. 2c). Visual deprivation induced an OD shift following magnesium treatment (Additional file 1: Figure S4b), and this shift was significantly reduced by the concurrent infusion of Ro 25-6981 (Fig. 2c, and Additional file 1: Figure S4b). Collectively, these findings indicate that NR2B-containing NMDARs play a key role in both the restoration and the expression of OD plasticity in adult mice.

### NR2B is not required to restore plasticity *via* a reduction in cortical inhibition

**Fig. 3:**
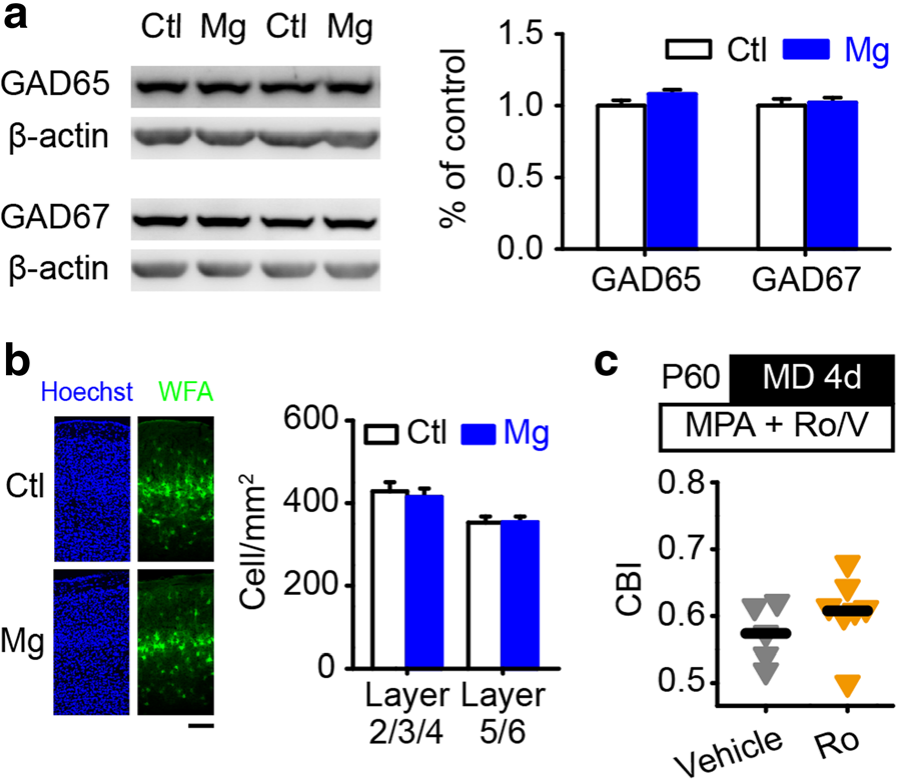
The distinct mechanism underlying the restoration induced by magnesium or reduced cortical inhibition. **a** The protein expression levels of GAD65 (Ctl, 17 mice; Mg, 15 mice) and GAD67 (Ctl, 12 mice; Mg, 11 mice) in V1b of control and magnesium-treated mice. Using two-tailed Student’s *t* test, P = 0.13 for GAD65, P = 0.73 for GAD67. **b** The density of cells surrounded by WFA-positive nets (green) in layers 2/3/4 and layers 5/6 of V1b in control (6 mice) and magnesium-treated (6 mice) mice. The DNA-binding dye Hoechst 33342 (Hoechst, blue) was used to identify the different layers. Scale bar, 100 μm. Using two-tailed Student’s *t* test, P = 0.65 for layers 2/3/4, P = 0.92 for layers 5/6. **c** Schematic of the experimental procedure (top) and a summary of the CBIs in adult MPA-treated mice with (Ro25-6981, Ro) or without (Vehicle, V) NR2B antagonist administration (bottom). The drugs were locally infused using an osmotic minipump one day prior to and concurrent with 4 days of MD. The horizontal bar represents the mean value; each symbol represents one animal. P = 0.26 using two-tailed Student’s *t* test. Error bar, SEM

It is well known that visual plasticity in adulthood can be restored by a reduction in cortical inhibition [25–28], which can be represented by a decrease in GABA-synthesizing enzymes or in perineuronal nets (PNNs) [15, 29, 30]. Interestingly, the protein expression of two major GABA synthetic enzymes (glutamate decarboxylase, GAD65/67) in the V1b of magnesium-treated mice was similar to that of control mice (Fig. 3a). PNN is a specialized form of the extracellular matrix that preferentially surrounds parvalbumin-positive inhibitory interneurons and participates in their maturation [29, 31]. PNN formation was determined by counting the density of cells surrounded by *Wisteria floribunda* agglutinin (WFA)-positive nets. PNNs were found to be abundant in layer 4, with no significant differences observed between control and magnesium-treated mice across the entire thickness of V1b (Fig. 3b). Thus, GADs and PNNs may not represent the predominant targets for magnesium treatment.

Dark rearing, which downregulates cortical inhibition and concurrently increases the expression of the NR2B subunit, is capable of restoring visual plasticity in adult rats [32]. To investigate whether the NR2B subunit is the downstream target regulated by a reduction in cortical inhibition, we concurrently infused Ro 25-6981 or vehicle, and the GABA synthesis inhibitor 3-mercaptopropionic acid (MPA) into V1b of adult mice (Fig. 3c). Consistent with previous study [27], the OD distribution in mice with MPA infusion shifted toward the open eye following MD (Additional file 1: Figure S4c). Furthermore, the NR2B antagonist failed to impair this shift in OD (Fig. 3c, Additional file 1: Figure S4c), indicating that different mechanisms underlie the restoration of plasticity *via* magnesium supplementation or a reduction in cortical inhibition.

### The rejuvenation of synaptic properties of layer 2/3 pyramidal neurons in V1b

**Fig. 4:**
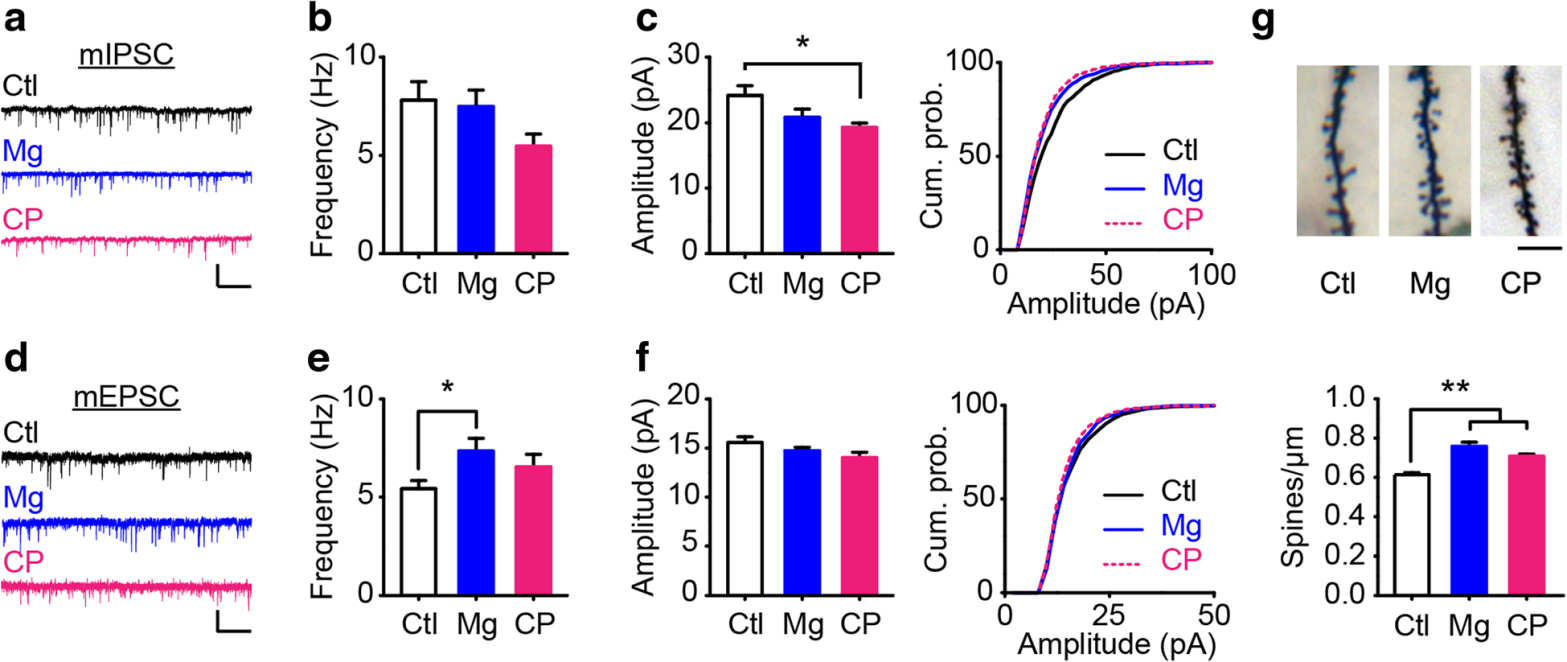
Juvenile-like synaptic properties following magnesium treatment. **a**, **d** Representative traces of mIPSCs and mEPSCs recorded from layer 2/3 pyramidal neurons in the visual cortex of adult control (Ctl), adult with magnesium treatment, and juvenile (CP; P25-P28, near the peak of the critical period) mice. Scale bars in (**a**), 50 pA and 1 s. Scale bars in (**d**), 25 pA and 1 s. **b**, **e** The mean frequencies of mIPSCs (**b**, Ctl, 20 cells in 6 mice; Mg, 20 cells in 6 mice; CP, 23 cells in 4 mice; Using Mann-Whitney *U* test, P = 0.08 for Ctl vs CP; P = 0.95 for Ctl vs Mg,) and mEPSCs (**e**, Ctl, 34 cells in 5 mice; Mg, 25 cells in 5 mice; CP, 24 cells in 4 mice; Using Mann-Whitney *U* test, P = 0.28 for Ctl vs CP; P = 0.03 for Ctl vs Mg). **c**, **f** The mean values and cumulative distributions of the amplitudes of mIPSCs (**c** left; Using Mann-Whitney *U* test, P = 0.007 for Ctl vs CP; P = 0.086 for Ctl vs Mg) and mEPSCs (**f** left; Using Mann-Whitney *U* test, P = 0.19 for Ctl vs CP; P = 0.61 for Ctl vs Mg). Using Kolmogorov-Smirnov test, P = 0.27 for CP vs Mg in (**c** right); P = 0.004 for CP vs Mg in (**f** right). **g** The density of dendritic spines of pyramidal neurons in layer 2/3 in Ctl (136 cells in 12 mice), Mg (80 cells in 10 mice) and CP (158 cells in 9 mice) group. Scale bar, 10 μm. P < 0.01 using a one-way ANOVA followed by Tukey’s *post hoc* test.*P < 0.05; **P < 0.01. Error bars, SEM

Both excitatory and inhibitory circuits, in addition to the morphology and density of dendritic spines in the visual cortex, undergo progressive maturation during development [8, 33–35]. We first measured miniature inhibitory and excitatory postsynaptic currents (mIPSCs and mEPSCs, respectively) from pyramidal neurons using whole-cell recording in layer 2/3 of acute visual cortical slices of juvenile and adult mice (Fig. 4a, d, Additional file 1: Figure S5a, b). The frequency and amplitude of mIPSCs were lower in juvenile mice than in adult mice. In contrast, the frequency of mEPSCs was slightly greater in juvenile mice than in adult mice, whereas the mean amplitude of mEPSCs was barely affected by age (Fig. 4a-f). Collectively, these findings indicate a functional shift of excitation/inhibition (E/I) balance during maturation. In adult mice, magnesium supplementation resulted in a strong increase in mEPSC frequency that was reminiscent of our observations in juvenile mice (Fig. 4e). The cumulative distribution of the amplitudes and inter-event intervals of mIPSCs and mEPSCs in magnesium-treated mice was similar to that of juvenile mice, although the mean values were not significantly different from those in untreated adult mice (Fig. 4b, c, f, Additional file 1: Figure S5c, d).

We further determined the density of dendritic spines on layer 2/3 pyramidal neurons in the visual cortex of above three groups. As expected, spine numbers in juvenile mice were significantly higher than that in normal adult mice (Fig. 4g), which was consistent with previous studies [8, 36]. Moreover, the spine density in magnesium-treated mice was increased by 23 %, which was comparable to that in juvenile mice (Fig. 4g). To further examine whether NR2B is also crucial to synaptogenesis following magnesium treatment, we locally infused NR2B antagonist Ro 25-6981 to V1b in one side of hemisphere and used V1b in the contralateral hemisphere as the control in magnesium-treated adult mice. Following blockade of NR2B activity, spine numbers exhibited a tendency of reduction, but it was not significant statistically (Additional file 1: Figure S6), suggesting that other signal pathways might involved in the regulation of synaptogenesis following magnesium treatment. Therefore, those findings imply that magnesium treatment adds functional excitatory synapses and rebalances the synaptic properties to a more juvenile-like state.

### Recovery of visual functions in adult amblyopia

**Fig. 5:**
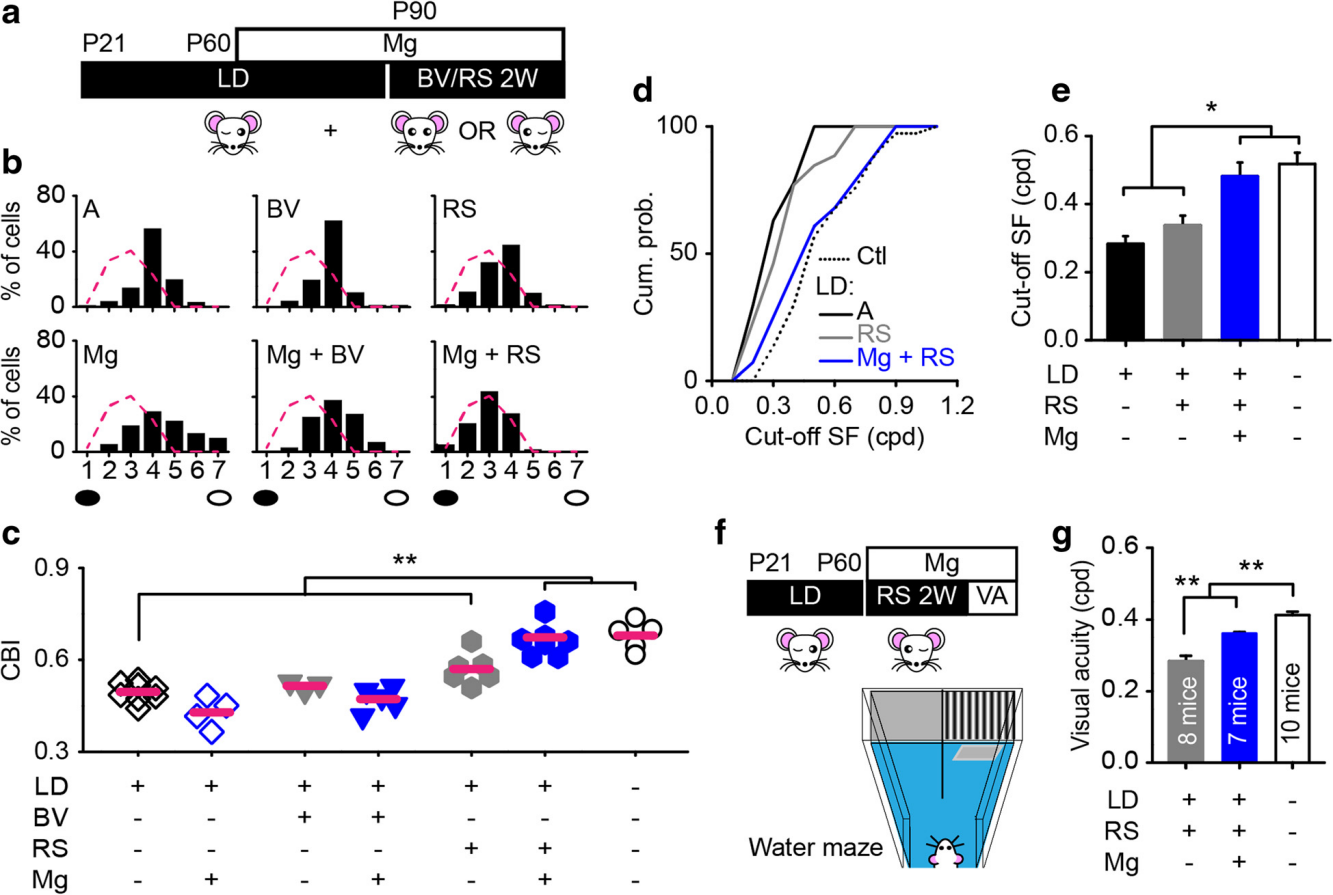
Magnesium facilitates the recovery of visual functions in adult amblyopic mice. **a** Schematic of the experimental procedure. 2 weeks of RS or BV were proceeded by 1 month of magnesium treatment in adult amblyopic mice. LD, long-term MD. **b** Comparison of the OD distribution in amblyopic group (A; CBI = 0.50 ± 0.01, 166 cells in 8 mice), amblyopic group with binocular vision (BV; CBI = 0.52 ± 0.01, 66 cells in 3 mice), reverse suturing (RS; CBI = 0.57 ± 0.02, 118 cells in 6 mice) or chronic magnesium treatment (Mg; CBI = 0.43 ± 0.03, 89 cells in 4 mice), and magnesium-treated amblyopic groups with binocular vision (Mg + BV; CBI = 0.7 ± 0.02, 99 cells in 5 mice) or reverse suturing (Mg + RS; CBI = 0.67 ± 0.02, 168 cells in 8 mice). The red dashed curve represents the OD distribution of normal adult mice shown in (Fig. 1b). Filled black circles represent the eye with LD, and open black circles represent the fellow eye. **c** Summary of the CBI values in each group. The horizontal bar represents the mean value. Each symbol represents one animal. **d**, **e** The cumulative distributions (**d**) and mean values (**e**) of the cut-off spatial frequency (cut-off SF) of V1b neurons driven by long-term deprived eye in amblyopic (27 neurons, 8 mice), reverse sutured amblyopic (26 neurons, 8 mice), magnesium-treated reverse sutured amblyopic (28 neurons, 8 mice) group, and either eye in control group (Ctl; 37 neurons, 5 mice). A and RS versus Ctl and Mg + RS, P < 0.01 using Kolmogorov-Smirnov test. **f** Schematic of the experimental procedure and the two-alternative forced-choice discrimination task. Visual acuity (VA) was measured following 2 weeks of magnesium treatment and RS. **g** Behavioral VA of the eye with LD in amblyopic mice and either eye in control mice. *P < 0.05, **P < 0.01 using a one-way ANOVA followed by Tukey’s *post hoc* test. Error bars, SEM

Amblyopia is a permanent impairment in spatial acuity and binocularity even after the correction of eye problems [37, 38]. The restoration of visual plasticity may facilitate the recovery of visual functions in adult amblyopia [3, 39, 40]. We sutured one eye in mice from P21 to adulthood to induce deprivation amblyopia (Fig. 5a). As expected, the contralateral eye dominance was impaired in untreated amblyopic mice, this deficit persisted even after 2 weeks of binocular vision (BV, Fig. 5b, c). Two weeks of reverse suturing (RS) could induce a mild recovery of the OD distribution (Fig. 5b, c), indicating that 2 weeks of visual alternation are long enough to elicit the residual plasticity in adult visual cortex [23, 41]. Next, we pretreated amblyopic mice with magnesium to reinstate the plasticity in adulthood prior to RS or BV (Fig. 5a). The CBI values of magnesium-treated amblyopic mice were slightly lower than those of untreated amblyopic mice, indicating an exacerbation of the deprived eye deficit (Fig. 5b, c). The combination of RS and magnesium supplementation significantly restored the CBI values and the OD distribution of amblyopic mice to normal levels, while magnesium treatment paired with BV was not effective in promoting recovery from amblyopia (Fig. 5b, c), suggesting that RS is an indispensable manipulation for the recovery.

We further measured the cut-off spatial frequency of V1b neurons in the same animal that was used to assess the OD distribution (Fig. 5d, e). Behavioral visual acuity was also determined using a two-alternative forced-choice discrimination task (Fig. 5f, g). We found that 2 weeks of RS was insufficient to improve visual acuity and the cut-off frequency of the formerly deprived eye (Fig. 5d-g). The behavioral visual acuity and the cut-off spatial frequency of V1b neurons driven by the long-term deprived eye, however, were recovered under the combination of RS and magnesium supplementation (Fig. 5d-g). Collectively, those results suggest that the plasticity rescued by magnesium treatment is effective in promoting the recovery of visual functions in adult amblyopic mice.

## Discussion

The E/I balance represents the functional barrier to limit plasticity in adulthood [3, 4, 6, 39]. In this study, we showed that both visual plasticity and synaptic properties of pyramidal neurons in the visual cortex was changed from adult to juvenile state following magnesium treatment. NR2B-containing NMDARs played a predominant role in the induction and expression periods of the magnesium-rescued plasticity, which was concurrent with an increase in glutamatergic excitation. However, the plasticity rescued by a reduction in cortical inhibition was intact following the blockade of NR2B-containing NMDARs. Therefore, our results revealed two distinct pathways underlying the resetting of E/I balance in adulthood.

The distinct property between juvenile and adult OD plasticity is whether cortical responses to the deprived eye are attenuated following brief MD [23, 24]. Manipulations such as environmental enrichment, dark rearing, fluoxetine administration or PSD-95 knockout restore a juvenile-like OD plasticity in adulthood [32, 42–44]. However, CNF-1 infusion or knockout of PirB, only enhance cortical responses to the non-deprived eye, indicating an adult-forms of OD plasticity [15, 16]. We restored a juvenile-like OD plasticity following magnesium treatment, without a potentiation of cortical responses to the non-deprived eye following 4 days of MD. This is consistent with a recent study in PSD-95 knockout mice [44]. One possible explanation to our result is that a reduction in NR2A/NR2B ratio is permissive for the non-deprived eye potentiation [24, 45, 46], while the NR2A/NR2B ratio was unaffected following magnesium treatment in our study. Moreover, chronic magnesium supplementation inhibits TNF-α expression [47], which is crucial to the homeostatic potentiation of cortical responses following MD [48].

Developmental maturation of synaptic function in the mouse visual cortex as well as its correlation with the restoration of OD plasticity in adulthood are still obscure. Our study demonstrated that the basal inhibitory synaptic transmission in layer 2/3 pyramidal neurons was strengthened beyond the critical period, consistent with the postnatal maturation of cortical inhibition suggested by previous studies [34, 49, 50]. We also observed a decrease in mEPSC frequency from the juvenile period to adulthood, which might result from a progressive reduction in total number of dendritic spines during postnatal development [8]. Magnesium treatment reinstated the OD plasticity, and compensated the reduction in spine density and mEPSC frequency. The change in mEPSC frequency is indicative of a presynaptic effect on the release process, such as alternation in release probability, number of synapses or release sites. Previous study have shown that elevation of extracellular magnesium concentration increases presynaptic release sites and puncta numbers, while decreases release probability in hippocampal slices of rats [19]. Thus, the release probability in the visual cortex may not be increased following magnesium treatment. Together, all findings suggest that the number of functional synapses is increased following magnesium treatment, which consequently form the structural basis for the rescued plasticity in adulthood [15, 16, 51]. Moreover, a juvenile states of synaptic properties and OD plasticity observed in V1b of magnesium-treated mice indicates the rejuvenation of adult visual cortex.

Maturation of cortical inhibition determines the onset and termination of critical period [52, 53]. Visual plasticity can be effectively elevated by a direct or indirect reduction in cortical inhibition [3, 4]. It is widely accepted that a reduction in cortical inhibition modulates E/I balance, which represents the functional barrier of plasticity in adulthood [3, 4, 54]. Interestingly, cortical inhibition also affects expression of molecular factors serve as the structural barrier of plasticity, such as perineuronal nets [27]. Therefore, cortical inhibition might modulate plasticity *via* structural and functional mechanisms. It is not surprising to observe the restoration of plasticity without a significant change in inhibition-related factors in our study. Several previous studies also enhanced plasticity without modulation of GAD65/67 and/or PNN levels in adult animals [15, 22, 30]. In most cases, i.e. dark rearing, the enhancement of serotonergic transmission with fluoxetine or the disinhibition of cholinergic signaling by knocking out Lynx1, a decrease in cortical inhibition as well as a potential increase in excitation may occur simultaneously [21, 32, 42]. Moreover, administration of the NMDAR co-agonist D-serine rescues OD plasticity and long-term depression in adult visual cortex [55]. Thus, the potentiation of glutamatergic excitation represents another effective strategy in reinstating the adult plasticity.

Specific NR2 subunits of NMDARs have distinct impact on receptor properties, synaptic plasticity, and disease [12, 56]. An increase in the expression of both NR2A and NR2B subunits is accompanied by an enhanced plasticity in the adult auditory cortex [57]. We observed a similar change in the expression of NR2 subunits following magnesium treatment. During early postnatal life, the predominance of NR2B represents the immature state of NMDARs [56, 58]. NR2B is also involved in multiple physiological processes, such as extracellular signal-regulated kinase 1/2 activation, AMPA receptor trafficking, synaptogenesis, all of which are essential to visual plasticity [14, 59, 60]. Moreover, both NR2B expression and visual plasticity are decreased following the critical period [9], indicating that NR2B-containing NMDAR represents another important factor for gating the termination of the critical period. Interestingly, the restoration of visual plasticity induced by reducing GABAergic inhibition was independent on the activity of NR2B-containing NMDARs. Hence, two pathways with different molecular mechanisms were revealed: (a) NR2B-dependent restoration primarily acting through an increase in glutamatergic excitation, and (b) NR2B-independent restoration induced by a direct reduction in GABAergic inhibition that may lead to elevated brain-derived neurotrophic factor expression and the subsequent activation of genes that regulate plasticity [42]. Due to the ineluctable limitation of pharmacological methods [61], more direct evidence associated with the contribution of NR2B to visual plasticity should be further investigated using NR2B conditional knockout mice [62].

Several therapies such as perceptual learning, video game playing and repetitive transcranial magnetic stimulation, have been proposed to improve visual acuity in adult amblyopic patients [63–65]. The underlying mechanism is still unclear, while the residual plasticity in the adult cortex seems to contribute to this recovery [63, 64, 66]. Indeed, plenty of investigations suggest that the enhancement of plasticity in adult visual cortex facilitates the recovery of amblyopia in adult rodents [5, 6, 40]. Our results further demonstrated that the rejuvenation of adult visual cortex with oral magnesium supplementation was effective in curing amblyopic mice with RS. Magnesium is a vital component of enzymatic reactions, second messenger pathway, energy metabolism, and neural plasticity [19, 67, 68]. Its supplementation has been used in therapies for numerous neurological disturbances, including depression, behavioral disturbances, seizures, ataxia, psychosis and aging [69–71]. It is plausible to concern that magnesium treatment is a nonspecific manipulation. Therefore, whether other pathways cooperate with NR2B-containing NMDAR-mediated pathway to rejuvenate visual cortex deserves further investigation.

## Conclusions

Here, our *in vivo* and *in vitro* results demonstrate that magnesium treatment facilitates the rejuvenation of the visual cortex in adult mice, and this restoration facilitates the recovery of both behavioral acuity and neural function in adult amblyopic mice. Our results further provide another direct evidence that the E/I balance is essential for the stabilization of cortical circuits in adulthood, while different mechanisms may be responsible for removing this functional barrier of plasticity. Collectively, we believe that NR2B-mediated signaling pathway may represent a novel approach to modulating cortical plasticity, and magnesium supplementation should be investigated further to develop a convenient and plasticity-based treatment for several human brain disorders, such as amblyopia, aphasia, schizophrenia and stroke [40, 43, 72, 73].

## Methods

### Animals

C57BL/6 mice were reared in standard cage, food and water were provided *ad libitum* and maintained on a 12 h:12 h light/dark cycle. Mice of either sex were used in this study. We preferred to use male mice for their better physical quality in electrophysiological experiments, and sex differences were not account for the observed effect in our studies. All procedures were approved by the Institutional Animal Care and Use Committee of the University of Science and Technology of China.

### Drug administration

Magnesium treatment was performed by administering magnesium L-threonate (MgT) *via* drinking water [19, 71]. Two doses of MgT (777 mg/kg/day for 1 month and 915 mg/kg/day for 2 weeks) were used in this study. The latter one is adapted to simultaneous administration of the NR2B antagonist Ro 25-6981 with an osmotic minipump (ALZET 1002) that can only last for 2 weeks. No significant difference was observed between mice that received these doses. MK801 (diluted with 0.9 % saline, 0.1 mg/kg, Sigma) was administered by intraperitoneal injection twice a day. PPPA (diluted with 0.01 M PBS, 0.083 mM, Tocris), TCN 201 (diluted with 0.01 M PBS, 100 μM, Tocris), Ro 25-6981 (diluted with 0.01 M PBS, 0.88 mM for 5 days of infusion (0.5 μl/h, ALZET 1007D) and 1.8 mM for 2 weeks of infusion (0.25 μl/h, ALZET 1002), Sigma) or MPA (diluted with 0.01 M PBS, 100 μM, Sigma) were directly infused into V1b using an osmotic minipump.

### Eyelid suture

MD was performed by suturing the eyelid of adult mice (> P90) under 0.5-3 % isoflurane anesthesia. To obtain amblyopic mice, long-term MD began at P21 and was maintained to adulthood (> P60). Following surgery, the mice were examined daily, and animals with spontaneous reopening of the sutured eye were excluded from subsequent experiments. Adult amblyopic mice were reverse sutured by opening the long-term deprived eye, whereas the fellow eye was sutured for 2 weeks. Chloramphenicol eye drops and cortisone eye ointment were used, and great care was taken to prevent any opacity of the eyes.

### Animal surgery for implantation

Mice were anaesthetized by inhalation of 0.5-3 % isoflurane and were placed in a stereotaxic frame for head fixation. Chlortetracycline eyeointment was applied to protect the eyes. Body temperature was maintained at 37 °C with a heating pad. Under aseptic conditions, after shaving and disinfecting the scalp, lidocaine was used for local anesthesia.

For electrode implantation, a small hole was drilled overlying V1b, and a microelectrode was inserted to 400 μm below the cortical surface, where the maximal amplitude of VEPs could be recorded [74]. The reference was a silver wire attached to a screw that was mounted in the contralateral prefrontal cortex.

Osmotic minipump implantation was performed as previously described [55, 75]. Briefly, the minipump was attached to the brain infusion cannula. A small hole (diameter = 0.5 mm) was drilled overlying the monocular region of the primary visual cortex at a position of 2 mm lateral to the midline and 1 mm anterior to the lambda; subsequently, the infusion cannula was inserted 1 mm below the surface of the skull. The attached minipump was placed in a subcutaneous pocket at the nape of the neck.

Following the implantation, the exposed skull was covered with cyanoacrylate and dental cement. Animals were allowed at least 4 days for recovery prior to subsequent experiments.

### *In vivo* electrophysiology

Mice were anaesthetized, maintained with a mixture of urethane (2 g/kg, i.p.) and chlorprothixene (5 mg/kg, i.m.) and placed in a stereotaxic frame. Body temperature was continuously monitored and maintained at 37 °C using a thermostatic electric blanket (Harvard). A craniotomy was performed over V1b, where recording was performed.

For single-unit recording, a computer-generated moving bar was presented on a monitor that was positioned 23 cm from the mouse’s eyes, cortical responses to each eye were recorded within the entire thickness of primary visual cortex. The mean firing rates of spontaneous and visually evoked activities were computed from peristimulus time histograms. In each mouse, 18–30 cells were recorded in three to six vertical penetrations that were evenly spaced (at least a 200 μm interval) across the mediolateral extent of V1b to avoid sampling bias. Only the cells with a receptive field within 20° from the vertical meridian were included in our sample. Cells were assigned to OD categories according to the seven-category scheme of Hubel and Wiesel [2]. ODs in the binocular zone of each mouse were calculated as a CBI, as follows: [(n_1_ - n_7_) + 2/3(n_2_ - n_6_) + 1/3(n_3_ - n_5_) + N]/2 N, where N = the total number of cells and n_x_ = the number of cells corresponding to an OD score of x. For the statistical comparison of OD distributions, normalized OD scores of single neurons were calculated using the following formula: (I – C)/(I + C), where C and I were the evoked contralateral and ipsilateral responses, respectively. To obtain the tuning curve of spatial frequency, moving sinusoidal gratings with different spatial frequencies were presented, and neurons were randomly sampled from the mice in which the OD distribution was recorded. Consistent with the method used to calculate spatial acuity recorded by VEPs [21, 42], the cut-off spatial frequency of a single neuron was obtained by extrapolation to the zero amplitude of the linear regression through the last four to five data points along a curve of the mean spike rate plotted against log spatial frequency.

For VEP recording, the visual stimuli were full-screen sinusoidal gratings of 100 % contrast, phase reversed at 2 Hz, and with a spatial frequency of 0.05 cycles/degree (cpd) presented on a monitor 23 cm from the eyes of anesthetized mice. A total of 100-200 visual stimuli were presented to each eye. Signals were band-pass-filtered (0.1–100 Hz), amplified, and fed to a computer for analysis. The VEP amplitude was quantified by measuring the peak-to-peak response amplitude.

### Cortical slice preparation and *in vitro* electrophysiology

Mice were deeply anaesthetized with sodium pentobarbital (83 mg/kg, i.p.), followed by transcardial perfusion with ice-cold oxygenated (95 % O_2_ and 5 % CO_2_) *N*-methyl-D-glucamine (NMDG) artificial cerebrospinal fluid (ACSF), which contained (in mM) 93 NMDG, 2.5 KCl, 1.2 NaH_2_PO_4_, 30 NaHCO_3_, 25 glucose, 20 HEPES, 0.5 CaCl_2_, 10 MgSO_4_, 3 glutathione (GSH), 5 sodium ascorbate, 3 sodium pyruvate and 2 thiourea (osmolarity: 295-310 mosmol, pH 7.2-7.3). After decapitation, the brains were rapidly removed, and cortical sections (250 μm thickness) containing V1b were cut in the same NMDG ACSF using a vibratome (Leica, VT1200S). Slices were immediately transferred to a recovery chamber containing 33 °C NMDG ACSF for 12-14 min and then transferred to 28 °C HEPES ACSF solution for > 45 min with the following composition (in mM): 92 NaCl, 25 KCl, 1.2 NaH_2_PO_4_, 30 NaHCO_3_, 25 glucose, 20 HEPES, 2 MgSO_4_, 2 CaCl_2_, 3 GSH, 5 sodium ascorbate, 3 sodium pyruvate and 2 thiourea (bubbled with 95 % O_2_ and 5 % CO_2_, 295-310 mosmol, pH 7.2-7.3). After a 1 h recovery period, recordings were performed at 31-32 °C in a recording chamber with constant normal ACSF flow (in mM, 129 NaCl, 3 KCl, 1.3 MgSO_4_, 1.2 KH_2_PO_4_, 20 NaHCO_3_, 10 glucose, 3 HEPES and 2.4 CaCl_2_, bubbled with 95 % O_2_ and 5 % CO_2_, 295-310 mosmol, pH 7.2-7.3).

Whole-cell voltage-clamp recordings were performed from pyramidal neurons of layer 2/3 of V1b using a HEKA EPC-9 amplifier (HEKA Electronics, Germany) with PATCHMASTER software and borosilicate recording pipettes with resistances of 2-6 MΩ. Electrical signals were filtered at 2.9 kHz and digitized at 10 kHz. Pyramidal neurons were identified by their morphology under an infrared differential interference contrast microscope (FN1, Nikon) and by their spiking pattern in response to depolarizing current (Additional file 1: Figure S5a). The neurons were held at -60 mV. For mIPSC recordings, the patch pipettes were filled with an internal solution consisting of (in mM) 120 KCl, 30 NaCl, 5 EGTA, 10 HEPES, 1 MgCl_2_, 0.5 CaCl_2_ and 2 Mg-ATP (pH 7.2, 285–295 mosmol). For mEPSC recordings, the solution contained (in mM) 130 Cs-methanesulfonate, 0.15 CaCl_2_, 2 MgSO_4_, 2 EGTA, 10 HEPES, 2 Na-ATP, 0.25 Na-GTP and 10 QX-314 (pH 7.2, 285–295 mosmol). Kynurenic acid (KYN; 4 mM) was added to the ACSF to eliminate excitatory components; 100 μM picrotoxin (PTX) was used to eliminate inhibitory components; and 1 μM tetrodotoxin (TTX) was added to eliminate spontaneous action potentials (Additional file 1: Figure S5b). Only cells with a series resistance < 30 MΩ and less than a 20 % change throughout the experiments were included for further study. Recordings were performed by examiners who were blinded to the treatment. Data analysis was conducted using Mini Analysis software (Synaptosoft). First 50 events/neuron were used to construct cumulative distribution of each group.

### Behavioral assessment of visual acuity

A two-alternative, forced-choice visual discrimination task in a visual water maze was used to behaviorally assess the visual acuity of mice [42, 76, 77]. Amblyopic mice learned the task during the period of RS, and their visual acuity was measured at the end of RS. The apparatus used in this study was identical to that used in a previous study [78], with the exception that only a 46 cm middle divider was used to set the choice line. Two sessions consisting of 20 trials were performed per day. First, the mice learned to associate a positive stimulus (a hidden escape platform) with a vertical low spatial frequency sinusoidal grating (0.12 cpd) and a negative stimulus (the absence of the escape platform) with a homogeneous grey (a mean luminance of 43 cd/m^2^). The location of the platform was pseudo-randomized with no more than three successive trials on an identical side. If the mouse broke the choice line to swim toward the gray arm, then the trial was considered an error. After the association was formed, the spatial frequency of the grating was progressively increased. If there were 4 consecutive correct choices in four successive trials or at least 6 correct choices in 8 consecutive trials, the spatial frequency of the grating was increased by a step of 0.03 cpd. If the accuracy decreased to below 70 % in 10 successive trials, the spatial frequency of the grating was decreased until a 70 % accuracy was achieved. The threshold for spatial frequency was repeatedly assessed several times. Finally, the accuracy for each spatial frequency was summarized to generate a frequency-of-seeing curve for each mouse. The visual acuity of the mouse was determined as the grating spatial frequency corresponding to 70 % accuracy.

### Western blot analysis

The primary visual cortex of adult mice was dissected under deep anesthesia with sodium pentobarbital (83 mg/kg, i.p.). The bilateral cortices of each mouse were mixed as one sample. Proteins were extracted with RIPA lysis buffer containing 0.01 M PBS (pH 7.4), 1 % NP-40, 0.5 % sodium deoxycholate, 0.1 % SDS, and complete EDTA-free protein inhibitor cocktail (Roche Applied Science). After blocking in 5 % nonfat dry milk (wt/vol, in 20 mM TBST), the membrane was incubated with rabbit anti-NR2A (1:1000, Abcam, ab77980), rabbit anti-NR2B (1:1000, Cell Signaling Technology, 4212S), rabbit anti-GAD65 (1:2000, Proteintech, 20746-1-AP), mouse anti-GAD67 (1:1000; Abcam, ab26116), and mouse anti-β-actin (1:15,000; Abcam, ab6276) antibodies, followed by the respective HRP-conjugated secondary antibodies (1:2500-1:10,000; Promega). The membrane was then developed using SuperSignal West Pico Chemiluminescent Substrate (Pierce). Western blot analysis was repeated multiple times, and the optical density of each band was determined using ImageJ software and normalized to that of β-actin.

### Immunohistochemistry

Mice were anaesthetized with sodium pentobarbital (83 mg/kg, i.p.) and then transcardially perfused with 0.9 % saline, followed by 4 % paraformaldehyde. The brain was postfixed in 4 % paraformaldehyde overnight and transferred to 30 % sucrose in 0.01 M PBS for 48 h. Coronal sections were cut at 30 μm thickness on a freezing microtome (Leica). Following treatment with 0.2 % Triton X-100 and blocking (2 % BSA in 0.01 M PBS) for 1 h, free floating sections were incubated with biotin-labeled lectin from *Wisteria floribunda* agglutinin (WFA, 1:200, Sigma, L1516) overnight at 4 °C. On the second day, the brain sections were rinsed with PBS and incubated with Alexa Fluor® 488-conjugated streptavidin (1:500, Molecular Probes, S11223) for 1.5 h at room temperature. After counterstaining with the nuclear dye Hoechst 33342 (2 μg/ml, Sigma, B2261) for 10 min to distinguish each sublayer, slides were mounted using Antifade Polyvinylpyrrolidone Mounting Medium (Beyotime). Subsequently, sections were imaged (10× objective) on a confocal microscope (LSM 710, Zeiss). All images were photographed using an identical gain, offset, and exposure time. PNNs were measured by counting the density of cells surrounded by WFA-positive nets in a 550 × 300 μm^2^ area (10 fields per mice) that covered the entire thickness of V1b. The counting was performed using Image-Pro Plus software (Media Cybernetics) by examiners who were blinded to the treatment.

### Golgi staining

Mice were deeply anaesthetized with sodium pentobarbital (83 mg/kg, i.p.) and perfused with 0.9 % saline. Brains were removed and transferred to Golgi–Cox solution, followed by 30 % (wt/vol) sucrose. Brains were then sectioned into 200 μm slices using a vibratome (Leica, Germany). Sections were mounted on polylysine-coated slides, developed, fixed, dehydrated, and coverslipped, and the neurons in V1b were observed. An optical microscope (Olympus, Tokyo) was used to image the labeled neurons under 64× magnification. For each mouse, at least 5–15 labeled typical pyramidal neurons in layer 2/3 of the binocular zone were randomly selected and analyzed using Image-Pro Plus software (Media Cybernetics) by examiners who were blinded to the treatment.

### Nissl staining

Following chronic recording of VEPs, mice with implanted microelectrodes were transcardially perfused with 0.9 % saline, followed by 4 % paraformaldehyde. The brains were removed and postfixed in 4 % paraformaldehyde overnight, followed by 30 % sucrose in 0.01 M PBS for 48 h. Coronal sections were cut into 30 μm sections on a freezing microtome (Leica) and then mounted on polylysine-coated slides. Following staining in Nissl solution (Beyotime), the slices were dehydrated, vitrified, coverslipped, and imaged using a stereoscopic microscope (SZX16, Olympus).

### Statistical analysis

We performed a two-tailed Student’s *t* test or Mann-Whitney *U* test to compare data sets from two independent experimental groups, paired Student’s *t* tests to compare data sets collected from an identical animal, and a one-way ANOVA followed by Tukey’s *post hoc* test to compare data sets from more than two independent experimental groups unless otherwise indicated. To compare the cumulative distributions, the Kolmogorov-Smirnov test was performed. Differences were considered significant at P values less than 0.05.

## Additional file

Additional file 1:
**Supplemental Figures S1-S6.** (PDF 562 kb)

## Abbreviations

NMDAR: *N*-methyl-D-aspartate receptor
OD: Ocular dominance
V1b: Binocular zone of the primary visual cortex
MD: Monocular deprivation
CBI: Contralateral bias index
VEP: Visually evoked potential
GAD: Glutamate decarboxylase
PNN: Perineuronal net
WFA: *Wisteria floribunda* agglutinin
mEPSC: miniature excitatory postsynaptic current
mIPSC: miniature inhibitory postsynaptic current
E/I: Excitation/inhibition
RS: Reverse suturing
BV: Binocular vision
MgT: Magnesium L-threonate
